# The Impact of Changing Social Support on Older Persons’ Onset of Loneliness During the COVID-19 Pandemic in the United Kingdom

**DOI:** 10.1093/geront/gnac033

**Published:** 2022-03-02

**Authors:** Athina Vlachantoni, Maria Evandrou, Jane Falkingham, Min Qin

**Affiliations:** Centre for Research on Ageing, Faculty of Social Sciences, University of Southampton, Southampton, UK; ESRC Centre for Population Change, Faculty of Social Sciences, University of Southampton, Southampton,UK; Centre for Research on Ageing, Faculty of Social Sciences, University of Southampton, Southampton, UK; ESRC Centre for Population Change, Faculty of Social Sciences, University of Southampton, Southampton,UK; ESRC Centre for Population Change, Faculty of Social Sciences, University of Southampton, Southampton,UK; ESRC Centre for Population Change, Faculty of Social Sciences, University of Southampton, Southampton,UK

**Keywords:** COVID-19, Relationship, Social isolation, Social networks, Well-being

## Abstract

**Background and Objectives:**

Social distancing measures aimed at controlling the spread of coronavirus disease 2019 (COVID-19) are likely to have increased social isolation among those older than 70 instructed to shield at home. This study examines the incidence of loneliness by gender over the first 10 months of the COVID-19 pandemic among persons aged 70 and older in the United Kingdom, and the impact of changing social networks and perceived social support on the new occurrence of loneliness.

**Research Design and Methods:**

Participants (*N* = 1,235) aged 70 and older with no reports of loneliness before the pandemic who participated in 7 rounds of the Understanding Society: COVID-19 Study (April 2020–January 2021) and the main Understanding Society Study conducted during 2019. Cox regression analyzed the time to a new occurrence of loneliness.

**Results:**

Among older people who hardly ever/never felt lonely before the pandemic, 33.7% reported some degree of loneliness between April 2020 and January 2021. Living in a single-person household, having received more social support before the pandemic, changes in support receipt during the pandemic, and a deteriorating relationship with one’s partner during the pandemic increased the risk of experiencing loneliness. Older women were more likely than older men to report loneliness, even when living with a partner.

**Discussion and Implications:**

During the 3 COVID-19-related lockdowns in the United Kingdom, changes in older people’s social networks and support resulted in a significant onset of loneliness. Findings highlight the risks of shielding older persons from COVID-19 in terms of their mental well-being and the importance of strengthening intergenerational support.

## Background and Objectives

Loneliness is “the unpleasant experience that occurs when a person’s network of social relations is deficient in some important way, either quantitatively or qualitatively” (Perlman & Peplau, 1981, p. 31). This concept relates to human health and well-being, which can affect psychological processes influencing physiological functioning, resulting in increased morbidity and mortality (Gale et al., 2018; Hawkley & Cacioppo, 2010; Jarach et al., 2021; Steptoe et al., 2013; Wenger et al., 1996). Strong social networks and receiving social support can be particularly important for older people (LaRocca & Scogin, 2015), alleviating loneliness and improving mental well-being (Chen et al., 2014).

On March 23, 2020, the United Kingdom went into lockdown in an attempt to limit the spread of coronavirus, with the Government mandating those who could do work at home, closing schools, restaurants, and all but essential shops, and advising individuals to stay at home and limit contact with individuals outside their household. On November 5, 2020 and January 6, 2021, respectively, second and third national lockdowns came into force in England. In addition to advice to the general population, the Government used the age cutoff of 70 years and over to define those who may be clinically vulnerable and need to take extra precautions including “shielding” at home (BBC News, 2020). Physical separation from one’s family and friends can make individuals more vulnerable if they are functionally dependent on relatives or specialized community services. Existing research shows that changes in persons’ relationships or their broader social network may lead to a suboptimal level of social interaction that can cause loneliness (Perlman & Peplau, 1981). For older people with reduced functional capacities, if support cannot be mobilized successfully, this may result in reduced physical and social interactions and feelings of disappointment or loneliness (Hwang et al., 2020).

Previous research has shown that socially isolated persons are at a greater risk of loneliness, although feelings of loneliness do not necessarily equate with feelings of social isolation (Klinenberg, 2016; Van Tilburg, 2021). Loneliness is a subjective feeling, reflecting the disparity between an individual’s desired and actual levels of social contact. The feeling of loneliness is never wanted and can take a long time to lessen. The feeling of social isolation is related to an individual’s number of social contacts or relationships. Individuals may prefer to have a small number of social contacts. Importantly, individuals may be able to overcome feelings of isolation relatively quickly by increasing their number of contacts (Care Connect & Age UK, 2018).

Recent research during the pandemic has highlighted the association between older people’s positive coping strategies and psychosocial well-being, showing that some individuals used unique adaptive mechanisms such as increased self-reflection to preserve their well-being during the pandemic (Minahan et al., 2021). Against this background, assessing the extent to which changes in older people’s social networks and support affected their mental well-being is a question of academic and policy significance.

This study uses a measure of loneliness that has previously performed well in general population surveys (Office for National Statistics [ONS], 2018; Vozikaki et al., 2018). It uses a direct question on how frequently respondents had felt lonely from the abbreviated version of the Center for Epidemiological Studies—Depression scale (Radloff, 1977) that is phrased as follows: “How often have you experienced the following feelings over the last week?”: “I felt lonely.” The question offered four response options (“almost all of the time,” “most of the time,” “some of the time,” and “almost none of the time”).

### Social Network, Social Support, and Loneliness

A social network includes all of an individual’s social contacts. Social support is typically divided into subtypes: emotional, instrumental, appraisal, and informational support (Berkman et al., 2000). Emotional support is related to love, sympathy, and understanding; instrumental support refers to help with needs such as getting groceries and cooking; appraisal support relates to helping in decision making or giving feedback; and informational support is about information provision.

Social networks and social support can significantly affect older persons’ health and functioning (Berkman et al., 2000). In the literature, these two terms are often used loosely and interchangeably. While social networks represent the number and quality of individuals’ relationships (Tomini et al., 2016); nevertheless, the structure and characteristics of social networks precede social support and potential benefits of social connection (Berkman et al., 2000). Social networks involving individuals outside the family offer emotional and instrumental social support and a sense of social engagement, positively affecting individuals’ mental and physical health (Stephens et al., 2011).

Research has shown that loneliness is influenced by the size of individuals’ social network, with those with fewer persons in their social network being more likely to feel lonely than those with a large social network (De Jong Gierveld, 1998; Dykstra et al., 2005; Moorer & Suurmeijer, 2001; Yang et al., 2020). Very old adults tend to have fewer social interactions and a smaller social network, reflecting the impact of life events that tend to happen later in life, such as retirement or the loss of loved ones (Wrzus et al., 2013). Moreover, the social network characteristics are crucial to understanding loneliness. A previous study found that older adults with locally integrated supportive networks with family, neighbors, and friends living nearby and high community involvement levels are most resilient (Wenger, 1997). Other studies found a link between being satisfied with one’s social network and loneliness (Kemperman et al., 2019), with older people living with a partner and feeling very close to him/her also reporting lower loneliness rates. Those with children but not feeling close to them reported higher rates of loneliness compared to childless persons (Demakakos et al., 2006). Household composition is also a critical determinant of loneliness (De Jong Gierveld & Van Tilburg, 1999), and those living with a partner are less lonely than those living alone (Demakakos et al., 2006).

### Gender and Loneliness

Empirical evidence has consistently challenged the stereotype that loneliness is more likely to be experienced by women (Maes et al., 2019; Victor et al., 2006). Older women are more vulnerable to loneliness as they live longer, are more likely to be widowed, to experience functional limitations, and to require more health care (Pinquart & Sörensen, 2001). However, divorce or widowhood in later life has a more adverse impact on the social life of men than women because men tend to focus on their partner as their main confidant, and most of their friendships have been dissolved by this age (Cooney & Dunne, 2001). Other research has shown that men are more likely to find an intimate attachment in marriages, while women tend to find protection from emotional loneliness in close ties outside marriage (Dykstra & de Jong Gierveld, 2004). In the United Kingdom, gender differences exist in the prevalence and correlates of loneliness, with women reporting more frequent feelings of loneliness than men (ONS, 2018). However, other research showed that gender was no longer independently associated with loneliness once the confounding influences of marital status, age, and living arrangement were excluded (Victor et al., 2006). A recent meta-analysis concluded that across the life span the mean levels of loneliness are similar for men and women (Maes et al., 2019). Therefore, the relationship between gender and loneliness remains unclear.

### The COVID-19 Pandemic and Loneliness

The coronavirus disease 2019 (COVID-19) pandemic has increased the risk of social isolation for many individuals, including older individuals. Recent research showed that changing social networks can affect older persons’ well-being (Vos et al., 2020), and that less contact with relatives links to higher loneliness (Hwang et al., 2020). It has also been shown that individuals with negative self-perceptions about aging and a stronger perception of themselves as a burden were more likely to experience loneliness (Losada-Baltar et al., 2021). At the same time, being a woman or living alone was associated with a higher risk of reporting greater loneliness during the pandemic (Seifert & Hassler, 2020). National-level data show that in Great Britain, about 5.0% of adults felt lonely often or always between April 3 and May 3, 2020 and were struggling to find things that help them cope during lockdown, which was a similar proportion to before the lockdown (ONS, 2020). A U.S. study found that a higher level of loneliness was associated with being under a “stay-at-home” order (Tull et al., 2020).

Among older adults with a worsening or severe loneliness, common reasons included insufficient social support and inadequate access to, or comfort with, social interaction technologies (Kotwal et al., 2020). During the pandemic, social isolation was exacerbated or initiated by the lockdown, while older adults’ access to formal and informal care networks was interrupted (Evandrou et al., 2020; Propper et al., 2020). Meanwhile, many older people are excluded from digital technology due to having low or no digital literacy or not having access (ONS, 2019). However, little is known about the incidence of loneliness and its relationship with changing social networks and social support among older people during the pandemic. In the current study, we seek to answer the following research questions: What are the risks and protective factors for the onset of loneliness over the observation period during the pandemic? Is there a gender difference in experiencing such loneliness? According to the above review, we hypothesize that in the context of the pandemic, a reduced social network is a risk factor, while receiving more social support is a protective factor against the onset of loneliness. Moreover, we hypothesize that older women are more likely to experience loneliness than men.

## Research Design and Methods

### Data

This study uses data from Waves 1–7 of the Understanding Society: COVID-19 Study conducted in 2020–2021 and the main Understanding Society data collected during 2019, with fieldwork taking place across the entire year (Institute for Social and Economic Research [ISER], 2021a). The UK Household Longitudinal Study (UKHLS; known as Understanding Society) is an ongoing nationally representative probability-based panel study of more than 40,000 households that began in 2009. Detailed information about the sampling methodology of UKHLS is described in the Understanding Society User Guides (ISER, 2021b, 2022). The Understanding Society COVID-19 Study started collecting data online immediately after the first COVID-19-related national lockdown in the United Kingdom. Between April 24 and April 30, 2020, all members of households who participated in either of the two most recent UKHLS data collections (Waves 8 or 9), and were older than 16 years, were invited to complete the first wave of the COVID-19 web survey. Those unable to make an informed decision due to cognitive impairments and those with unknown postal addresses or addresses abroad were excluded. The first round of the COVID-19 survey was fielded between April 24 and April 30, 2020 (*n* = 16,662), the second round between May 27 and June 2 (*n* = 14,607), the third between June 25 and July 1 (*n* = 13,917), the fourth between July 24 and July 31 (*n* = 13,577), the fifth wave between September 24 and October 1 (*n* = 12,696), the sixth wave between November 24 and December 1 (*n* = 11,802), and the seventh wave between January 27 and February 3 (*n* = 11,797). The response rate (full interview) of seven waves of the Understanding Society COVID-19 Study was 39%, 35%, 33%, 32%, 30%, 28%, and 28%, respectively (ISER, 2021b).

Given our focus on the *incidence* of loneliness, all older people aged 70 and older who reported feeling lonely “hardly ever or never” before the pandemic, who participated in all seven waves of the COVID-19 Study and had no missing data on loneliness were included in the study. In Wave 1 of the COVID-19 Study (April 2020), a total of 1,931 older people aged 70 and older who had hardly ever or never felt lonely before the pandemic participated in the survey. During the subsequent six waves of the COVID-19 Study, 696 respondents were lost to follow-up, resulting in a final analytical sample of 1,235 respondents and 8,645 observations. The characteristics of the sample are given in Table 1. The analytical sample was slightly younger, wealthier, and more likely to live with a partner, but less likely to live in a single-person household at Wave 1 of the COVID-19 Study than those lost to follow-up. No difference was observed with regard to the receipt of practical help, emotional support, financial transfers, and the closeness in one’s partner relationship (Supplementary Table 1).

**Table 1.** Social Support (living arrangements, instrumental, emotional, and financial support during the pandemic) Among People Aged 70 and Older and the New Occurrence of Loneliness (*N* = 1,235)

Respondent characteristics	Total sample			New occurrence of loneliness^a^		
	%	*M* (*SD*)	*n*	%	*M*	*p*
Total	100.0		1,235	33.7		
Living arrangements						<.001
With adults or others	18.3		210	33.8		
Single-person household	15.6		179	55.2		
With partner 70+ only	53.4		689	26.2		
With partner younger than 70 only	12.8		157	38.8		
Practical help receipt from family, neighbors, or friends outside the household						<.001
No	34.7		447	21.9		
Yes	65.3		788	40.0		
Change in practical help receipt						<.001
No change	53.7		650	28.8		
More help received or received help from someone who did not previously help me	40.9		515	39.4		
Less help received	2.5		32	23.8		
Other	2.8		38	47.8		
Prepandemic emotional support from outside the household						<.001
A lot	19.1		245	52.6		
Some	31.6		382	34.1		
A little	28.0		325	30.1		
None	21.3		283	20.8		
Change in emotional support from outside the household						<.001
More	16.7		203	51.9		
About the same	76.0		937	28.3		
Less	7.3		95	47.5		
Score of prepandemic contact (including face to face, by phone, and virtual contact with people outside the household)		11.9 (3.5)	1,235		11.7 for nonlonely group 12.2 for lonely group	.08
Score of contact after the pandemic (including face to face, by phone, and virtual contact with people outside the household)		10.1 (3.6)	1,235		10.1 for nonlonely group 10.3 for lonely group	.303
Financial transfer						.069
No transfer	85.4		1,027	32.6		
Has transfer	14.6		208	40.2		
Partner relationship (closeness) change						.001
About the same or not in a relationship	91.7		1,123	33.6		
Better than before	7.3		97	27.1		
Worse than before	1.0		15	87.5		

*Notes:* Source: Authors’ analysis, Understanding Society: COVID-19 Study, 2020–2021. All proportions are weighted using longitudinal sample weights. Number of respondents is unweighted. For significance tests, analysis of variance tests were used for the association between loneliness and numerical variables, including the mean score of prepandemic contact (measured in 2019) and mean score of contact during the pandemic. Chi-square tests were used for the association between loneliness and all other categorical variables.

^a^Respondents were asked how frequently they had felt lonely in the last 4 weeks and were offered three response categories: (a) hardly ever or never, (b) some of the time, and (c) often. We grouped the latter two categories and generated a new binary variable including the categories of feeling lonely sometimes or often (coded as 1), and hardly ever or never (coded as 0), at each wave of the survey.

### Measurement

#### Time to event (loneliness)

After 2019, the respondents were assessed at every wave of the COVID-19 Study, which was in Wave 1 (April 2020), Wave 2 (May 2020), Wave 3 (June 2020), Wave 4 (July 2020), Wave 5 (September 2020), Wave 6 (November 2020), and Wave 7 (January 2021). The outcome of interest was a new occurrence of loneliness. In order to assess this outcome, respondents were asked how frequently they had felt lonely in the last 4 weeks and were offered three response categories: (a) hardly ever or never, (b) some of the time, and (c) often. We grouped the latter two categories as only a few older people reported feeling lonely often (among 1,235, only 4.0% felt lonely often at least once between April 2020 and January 2021, and 29.7% felt lonely some of the time). This generated a new binary variable including the categories of feeling lonely sometimes or often (coded as 1), and hardly ever or never (coded as 0), at each wave of the survey. The time to event was calculated as the number of waves the respondent participated in before reporting loneliness or the last wave follow-up if no event occurred and ranged from 1 to 7.

*Social networks and social support* were measured using a range of variables. The respondents’ living arrangement differentiated between living in a single household, with a partner aged 70 and older only, with a partner younger than 70 only, or with another adult(s). The survey also enquired about practical help received from one’s family, neighbors, or friends after the pandemic (yes, no); and perceived changes in such support since the pandemic (more, less, about the same), and similarly about the receipt of emotional support before the pandemic (a lot, some, little, none) and perceived changes in such support since the pandemic (more, less, about the same). All questions about the receipt of support referred to support received from individuals outside the respondents’ household.

A contact score before and after the pandemic was computed using two continuous variables. Respondents rated each of three items before and after the pandemic (face to face, phone call, or virtual contact) on a 7-point Likert-type scale corresponding to the following categories: daily, several times per week, at least once per week, several times per month, at least once per month, less often, and never. Items were summed to form two total contact scores (before and after the pandemic), potentially ranging from 0 to 18, with a higher score indicating more frequent contact. Finally, respondents were asked about having received or provided financial help after the pandemic to family/ friends who do not live in the same house (yes, no).

#### Demographic, socioeconomic characteristics, and adverse health conditions

The respondents’ demographic characteristics in the analysis included gender (male/female) and age group (70–74, 75–79, and 80+). We did not include race/ethnicity as a demographic variable as more than 95% of the analytical sample (1,183) are White British, and only 52 are non-White British. Measuring socioeconomic status in older age groups presents challenges (Grundy & Hold, 2001). Because most of the study respondents have retired, occupation and income might not be a good differentiator. Educational attainment is usually fixed early in life, but many of the older population in this survey data set left school at a young age with no qualifications (Grundy & Hold, 2001). Given these limitations, the respondents’ socioeconomic background was measured here using their housing tenure (own outright, own with mortgage, and rent).

Adverse health condition measures included the number of functional limitations in activities of daily living (ADLs) and instrumental activities of daily living (IADLs; none, one limitation, two or more limitations) and three categories on the report of a long-term health condition (no long-term health condition; emotional, nervous, or psychiatric problem; other long-term health condition). The health measures referred to the time before the pandemic.

In order to capture any period effects associated with the evolution of the pandemic, we controlled for the survey time point measured as a dummy variable (April, May, June, July, September, November, and January).

### Analytical Approach

Survival analysis using Cox regression was used to model the time from the 2019 baseline until a report of loneliness or until the end of the follow-up period if participants had no loneliness reported. Sensitivity analyses based on binary outcomes with mixed-effects logistic models then assessed the associations between loneliness and changing social support. Given that the data collected from individual respondents between April 2020 and January 2021 are not independent of each other, mixed-effects models were used to take account of between- and within-individuals variance (StataCorp, 2019); such models have been previously used to analyze the longitudinal relationship between loneliness, social isolation, and frailty in older adults (Davies et al., 2021). Moderation effects were tested by introducing interaction terms. The analyses were carried out in STATA version 15 (StataCorp, 2019).

Key independent variables, as noted above, included social networks, social support (e.g., living arrangements, practical help received from outside the household after the pandemic, and changes since the pandemic), emotional support before and changes since the pandemic, contact score before and after the pandemic, and financial transfers after the pandemic. Other potential confounding demographic, socioeconomic, and adverse health variables included the respondents’ age, gender, housing tenure, the number of ADL and IADL limitations, the report of long-term health conditions, and, for those who had a partner, the perceived change in the closeness of the relationship with one’s partner before and during the lockdown. Given research on the differential benefits of marriage between men and women (Dykstra & de Jong Gierveld, 2004), we included an interaction between gender and whether the respondents coresided with their partner. The results of the analysis are presented as hazard ratios (HRs) of reporting loneliness.

## Results

Table 1 provides an overview of the characteristics of the analytical sample along with bivariate analysis of new occurrences of loneliness. Among older people aged 70 and older who hardly ever/never felt lonely before the pandemic, a third (33.7%) reported onset of loneliness during the pandemic; 29.7% felt lonely sometimes and 4.0% felt lonely often. The majority of the sample lived with a partner (66.2%), although one in six older people who hardly ever/never felt lonely before the pandemic lived in a single household during the pandemic. Just under two thirds (65%) reported receiving instrumental care from outside the household. Since the pandemic, 41% of older people had received or were receiving more care from someone who did not provide care to them before the pandemic, and only 3% received less care. After the pandemic started, 17% of older people received more emotional support, while 7% received less. Compared with before the pandemic, older people reported much less face-to-face contact, whereas contact through phone calls or virtually remained at a similar level. For almost all of these characteristics, there was a significant bivariate relationship with the likelihood of reporting a new occurrence of loneliness (right-hand column, Table 1), with over half of those living alone (55.2%) doing so compared to just over a quarter (26.2%) of those living with a partner aged 70 and older.

**Figure 1.**
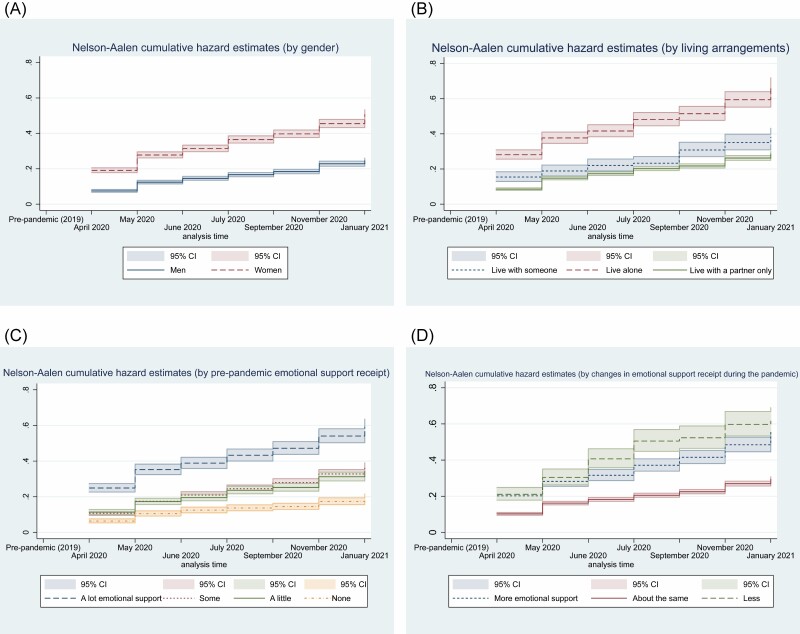
Cumulative hazard curves by (**A**) gender, (**B**) living arrangements, and social support receipt (**C**) pre-pandemic and (**D**) change during pandemic.

Cumulative hazard curves showing the cumulative incidence of loneliness among older men and women and different living arrangements are presented in Figure 1. Women had a higher cumulative hazard of loneliness than men; for women, the cumulative incidence by January 2021 was 51% (95% confidence interval [CI] = 48%–53%), the corresponding figure for men was 25% (95% CI = 23%–26%). Older people living alone had the highest cumulative hazard of feeling lonely during the pandemic, rising to 67% by January 2021 (95% CI = 62%–72%). By comparison, the figure for those living with a partner only was 29% (CI = 27%–30%). Those receiving more emotional support or having more contact with people outside the household prepandemic also experienced a higher hazard of postpandemic loneliness over time. Further information on other characteristics is presented in Supplementary Table 2. A greater proportion of older people who had financial transfers reported a new occurrence of loneliness. By contrast, those who reported a better relationship with their partner than before the pandemic had a lower chance of reporting loneliness over time (Supplementary Table 2).

Many of the characteristics discussed so far, such as gender and living arrangements, may be correlated. Table 2, therefore, presents multivariate analysis of the adjusted HR of loneliness. Model 1 shows the main effects among all respondents, while Model 2 includes additional interaction terms of gender and living arrangements. Given the significance of these, Models 3 and 4 present the results of separate models for older men and women.

**Table 2.** Estimated HRs and 95% CI From Cox Models Among All Respondents, Men and Women

Respondent characteristics	Model 1 (main effects)	Model 2 (with interaction terms)	Model 3 (among men)	Model 4 (among women)
	HRs (95% CI)	HRs (95% CI)	HRs (95% CI)	HRs (95% CI)
Living arrangement (ref: With an adult)				
Single-person household	1.34*** (1.16–1.55)	1.42** (1.14–1.78)	1.41** (1.13–1.77)	1.59*** (1.30–1.94)
With partner aged 70+ only	0.67* (0.58–0.77)	0.48*** (0.39–0.59)	0.45*** (0.37–0.56)	0.95 (0.78–1.16)
With partner younger than 70+ only	0.92 (0.77–1.10)	0.72** (0.58–0.91)	0.66*** (0.52–0.83)	1.36* (1.01–1.82)
Receipt of practical help after the pandemic (ref: No)				
Yes	1.28*** (1.16–1.42)	1.29*** (1.17–1.43)	1.71*** (1.47–1.99)	1.07*** (0.94–1.22)
Change in receiving practical help (ref: No change)				
Increase	0.96 (0.87–1.05)	0.97 (0.88–1.06)	0.93 (0.81–1.07)	1.06 (0.94–1.21)
Decrease	0.88 (0.69–1.11)	0.90 (0.71–1.14)	0.82 (0.53–1.28)	1.02 (0.66–1.36)
Prepandemic emotional support (ref: A lot)				
Some	0.71*** (0.64–0.77)	0.70*** (0.63–0.77)	0.77** (0.64–0.92)	0.67*** (0.59–0.76)
A little	0.75*** (0.67–0.84)	0.74*** (0.67–0.83)	0.70*** (0.57–0.85)	0.76*** (0.66–0.87)
None	0.52*** (0.45–0.60)	0.51*** (0.45–0.59)	0.55*** (0.45–0.69)	0.50*** (0.41–0.61)
Change in emotional support (ref: About the same)				
More	1.44*** (1.30–1.58)	1.45*** (1.31–1.59)	1.83*** (1.55–2.17)	1.33*** (1.18–1.50)
Less	1.98*** (1.75–2.24)	2.01*** (1.77–2.27)	3.12*** (2.62–3.72)	1.38** (1.15–1.66)
Prepandemic contact score	1.02* (1.00–1.03)	1.02* (1.00–1.03)	1.03** (1.01–1.06)	1.01 (0.99–1.03)
Contact score after the pandemic	0.99† (0.97–1.00)	0.99† (0.97–1.00)	0.99 (0.97–1.01)	0.98 (0.97–1.00)
Financial transfer after the pandemic (ref: No)				
Yes	1.83*** (1.17–1.41)	1.30*** (1.18–1.42)	1.48*** (1.27–1.71)	1.20** (1.06–1.36)
Partner relationship change (closeness; ref: No change or not in relationship)				
Better than before	0.94 (0.81–1.10)	0.93 (0.80–1.09)	1.03 (0.82–1.31)	0.81† (0.65–1.00)
Worse than before	3.43*** (2.69–4.39)	3.59*** (2.81–4.58)	3.89*** (2.79–5.41)	3.07*** (2.10–4.51)
Age group (ref: 70–74)				
75–79	1.12* (1.02–1.22)	1.13** (1.03–1.23)	0.78** (0.67–0.91)	1.35*** (1.21–1.51)
80+	1.05 (0.94–1.18)	1.07 (0.96–1.20)	0.96 (0.81–1.15)	1.08 (0.92–1.26)
Gender (ref: Men)				
Women	1.66*** (1.52–1.81)	1.14 (0.89–1.46)		
Housing tenure (ref: Own outright)				
Owned with mortgage	0.79* (0.63–0.99)	0.80* (0.64–0.99)	0.36*** (0.25–0.53)	1.57** (1.18–2.10)
Rent and other	0.99 (0.86–1.16)	1.01 (0.87–1.18)	0.86 (0.64–1.16)	1.09 (0.91–1.29)
Number of ADL and IADL difficulties (ref: None)				
1	1.32*** (1.18–1.47)	1.32*** (1.18–1.48)	1.65*** (1.38–1.98)	1.25** (1.08–1.45)
2+	1.33*** (1.15–1.53)	1.30*** (1.13–1.51)	1.16 (0.90–1.50)	1.44*** (1.20–1.71)
Long-term health condition (ref: No)				
An emotional, nervous, or psychiatric problem	1.03 (0.78–1.37)	1.05 (0.79–1.40)	0.62 (0.29–1.33)	1.15 (0.84–1.58)
Other long-term health condition	1.12* (1.01–1.23)	1.11* (1.01–1.22)	1.51*** (1.28–1.80)	1.00 (0.89–1.13)
Month (ref: April)				
May	1.00 (0.87–1.16)	1.00 (0.87–1.16)	1.04 (0.83–1.31)	0.98 (0.82–1.18)
June	1.00 (0.87–1.16)	1.00 (0.87–1.16)	1.03 (0.82–1.29)	0.98 (0.82–1.18)
July	1.01 (0.88–1.17)	1.02 (0.88–1.17)	1.05 (0.84–1.32)	0.99 (0.82–1.19)
September	1.01 (0.88–1.17)	1.02 (0.88–1.17)	1.07 (0.85–1.34)	0.98 (0.82–1.18)
November	1.10 (0.95–1.28)	1.11 (0.96–1.29)	1.30* (1.02–**1.64)**	1.00 (0.83–1.22)
January	1.10 (0.95–1.27)	1.11 (0.95–1.28)	1.28* (1.01–**1.62)**	1.00 (0.83–1.21)
Living arrangement × Gender				
Women × Single-person household		1.00 (0.75–1.35)		
Women × With partner aged 70+ only		1.79*** (1.37–**2.35)**		
Women × With partner younger than 70+ only		1.67** (1.16–**2.41)**		
Number of observations	8,645	8,645	4,760	3,885
Model fit log likelihood	−23,302.28	−23,280.44	−8,531.13	−23,280.44
LR test *p* value	<.001		<.001	<.001

*Notes:* HR = hazard ratio; CI = confidence interval; ADL = activities of daily living; IADL = instrumental activities of daily living. Source: Authors’ analysis, Understanding Society: COVID-19 Study, 2020–2021. Including the interaction terms creates a statistically significant improvement in the fit of the model.

***p < .001, **p < .01, *p < .05, †*p* < 0.1.

The results confirm many of the findings so far. Significant higher hazards are observed for women (HR = 1.66; 95% CI: 1.52–1.81), those living in a single-person household (HR = 1.34; 95% CI: 1.16–1.55), receiving a higher level of prepandemic emotional support or contact score (HR = 1.02; 95% CI: 1.00–1.03), and those receiving more or less emotional support during the pandemic (HR = 1.44, 1.98; 95% CI: 1.30–1.58, 1.75–2.24). Moreover, older people whose relationship with their partner had worsened during the pandemic exhibited a higher hazard of loneliness (HR = 3.43; 95% CI: 2.69–4.39). Those with ADL or IADL limitations (HR = 1.32, 1.33; 95% CI: 1.18–1.47, 1.15–1.53) and those with a long-term health condition (HR = 1.12; 95% CI: 1.01–1.23) showed a higher hazard of feeling lonely (Model 1 in Table 2). The incidence of loneliness was relatively stable across the whole period, with no reduction at the end of the first lockdown in summer 2020, although there is some sign of an increase in November 2020 and January 2021, coinciding with the second and third national lockdowns.

Model 2 in Table 2 added an interaction term between gender and whether the respondents coresided with their partner. The results highlight that the strength of the association between coresiding with one’s partner and a new occurrence of loneliness differs by gender. Women were more likely than men to report loneliness, even when living with a partner (HR = 1.79, 1.67; 95% CI: 1.37–2.35, 1.16–2.41).

Separate models for men (Model 3) and women (Model 4) show gender differences, for example, living with a partner lowers the hazard of loneliness among men (HR = 0.45, 0.66; 95% CI: 0.37–0.56, 0.52–0.83) but increases the hazard among women (HR = 1.36; 95% CI: 1.01–1.82; Table 2). Social networks and social support variables had similar HRs among men and women. Compared to the age group 70–74, men aged 75–79 had a lower hazard, while women in this age group had a higher hazard. Owning a house with a mortgage lowered the hazard among men, but increased the risk among women. Long-term health conditions and survey months November and January increased the hazard among men, but not women.

Similar patterns are found using the mixed-effects models, with sensitivity analyses providing further confidence that the results and observed differences are robust (Supplementary Table 3).

## Discussion and Implications

Existing research in the United Kingdom has highlighted the importance of investigating the prevalence of loneliness among older people with different characteristics (Demakakos et al., 2006; ONS, 2018; Victor et al., 2002). During the first UK lockdown, the government identified those aged 70 and older as “clinically vulnerable,” regardless of medical conditions, and encouraged them to minimize contact with anyone outside their household. This study examined the incidence of loneliness and its correlates over 10 months during the pandemic among older people aged 70 and older. To our knowledge, this study is the first to examine the new incidence of loneliness among older persons during the pandemic. The first key result is that among older people who hardly ever/never felt lonely before the pandemic, between April 2020 and January 2021, one third reported onset of loneliness, although the majority among those felt lonely “sometimes” (29.7%), and 4% felt lonely “often.”

In addition, those who received more social support from outside the home prior to the pandemic and who experienced changes in such support had a higher incidence of loneliness. This might be because older people who received more emotional support before the pandemic had higher support needs, and during the pandemic, this subgroup may then have experienced a greater level of need. We were not able to directly measure such unmet needs; however, this is an important avenue for future research. Those whose relationship with their partner worsened during the pandemic showed a higher loneliness risk. Recent research found that maintaining social communication amidst social distancing measures created a “buffer” for older adults’ well-being in Switzerland (Macdonald & Hülür, 2021). Practical, emotional, and financial support are often related to mental well-being in general (Manuel et al., 2012). However, the pandemic has also affected older persons’ social networks, leading to the new experience of loneliness. Although more financial transfers imply more interaction with others, older people are more likely to give out rather than receive such support, and outflows of financial support and related stress might increase the incidence of loneliness.

A second key finding is that older women were more likely than men to feel lonely during the pandemic, a result that is consistent with other studies (ONS, 2020; Pinquart & Sörensen, 2001; Vozikaki et al., 2018). One explanation is that women consider interpersonal relations to be more critical to their own well-being than men (Borys & Perlman, 1985), and deficiencies in their social relationships or changes may be more likely to be noticed and to have adverse effects for women. Our study also showed that the strength of the association between partner coresidence and loneliness differs by gender, with women living with a partner being more vulnerable. This may be due to married men and women being differentially susceptible to loneliness, as previous research has shown women placing less emphasis on their marriage than men as a way of developing and maintaining social ties (Dykstra & de Jong Gierveld, 2004). During the pandemic, social contact with individuals outside the household dramatically reduced, and this could have affected women more than men, even women coresiding with a partner.

Finally, the study shows that the hazards of experiencing loneliness did not significantly reduce over the 10-month observation period. Previous research found that older adults may respond to gaps in their social support through different channels, for example, turning to substitute sources of support or redefining their social goals (Rook, 2009), which, in turn, might attenuate the feeling of loneliness. However, our results seem not to provide such evidence. Our results are consistent with a recent UK study (Bu et al., 2020) showing that levels of loneliness during the lockdown were established early in the lockdown period and were then relatively stable over time. These levels were high compared to before the pandemic, with no signs of improvement, showing little evidence either of adaptation of loneliness responses to the circumstances during the pandemic, or growing sensations of loneliness, which may be due to the pandemic’s ongoing and global effect (Bu et al., 2020). Understanding the nuances of such adjustments to older adults’ changes in their social support resources, both during the pandemic and beyond, is beyond the scope of this article.

The study includes a number of limitations. Firstly, our study was restricted to participants aged 70 and older with no reports of loneliness before the pandemic. Those who were unable to make an informed decision due to cognitive impairments were ineligible for the COVID-19 survey. It is likely that some of these individuals would have experienced increased social isolation during the pandemic given that cognitively impaired individuals may have difficulties maintaining friendships or communicating with others (Brown et al., 2011). As such, the overall level of loneliness onset might be underestimated in our analysis. Additionally, over the observation period, nearly 700 respondents were lost to follow-up, which may bias our results. The supplementary results suggest that some of those lost to follow-up may have been more likely to experience social isolation and loneliness. A further limitation is the low number of respondents from Black and other minority ethnic groups in the sample, meaning ethnicity could not be included in the analysis. Finally, questions about the receipt of financial support were limited to the later waves, rather than comparing with before the pandemic. It is important for future studies to examine changes in all kinds of support before, during, and after the pandemic.

In conclusion, this is the first study, to our knowledge, of the impact of changes in older people’s social networks and social support during the UK lockdown on the onset of loneliness. Our analysis showed that among older persons who had hardly ever or never experienced loneliness before the pandemic, approximately one third had experienced loneliness during the pandemic, and the initial onset of loneliness may then have persisted for approximately 10 months. It is essential to consider how to tackle loneliness during the pandemic among older persons. Promoting digital technologies to bridge the social distance and the development of outreach and screening for loneliness alongside associated mental health conditions might be helpful (Galea et al., 2020). Such initiatives may help as more contact protects against loneliness. However, our results suggest that only increasing certain types of support, such as emotional support, might be insufficient to tackle loneliness. Notably, loneliness during COVID-19 has been associated with poorer mental health (ONS, 2020), suggesting that there may be an exacerbation of worries among lonely persons. Therefore, strategies addressing loneliness may require greater nuance beyond providing extra social support. In addition, gender differences permeate the risk, with women being more likely to experience a new occurrence of loneliness than men, even those coresiding with a partner. Therefore, interventions to tackle loneliness should integrate a gender lens. Taken together, these findings highlight the risks of segmenting and shielding older persons from COVID-19 in terms of their mental well-being (Iacobucci, 2020). More efforts to strengthen intergenerational solidarity during the pandemic can directly benefit older people’s mental well-being (Ayalon et al., 2021).

## Supplementary Material

gnac033_suppl_Supplementary_Material
