# Supplementary material for: The Impact of Changing Social Support on Older Persons’ Onset of Loneliness During the COVID-19 Pandemic in the United Kingdom
Source: Gerontologist. 2022 Mar 2;62(8):1147–59. doi: 10.1093/geront/gnac033 (PMC8903469; doi:10.1093/geront/gnac033)
Supplement: gnac033_suppl_Supplementary_Material [file gnac033_suppl_supplementary_material.docx]

**Supplementary Table 1. Characteristics of full wave 1 sample and analytical (follow-up) sample**

| **Respondent Characteristics** | **Full wave 1 sample** | **Analytical sample** | **p*** |
| --- | --- | --- | --- |
| **Number of respondents** | 1931 | 1235 |  |
| **Age, mean (SD)** | 76.1 (5.0) | 75.9 (4.5) | 0.003 |
| **Age** |  |  | 0.001 |
| 70-74 | 45.5 | 48.0 |  |
| 75-79 | 31.0 | 31.9 |  |
| 80+ | 23.5 | 20.1 |  |
| **Gender** |  |  | 0.340 |
| **Men** | 55.9 | 56.9 |  |
| **Women** | 44.1 | 43.1 |  |
| **Housing tenure** |  |  | <0.001 |
| Own outright | 81.7 | 87.7 |  |
| Owned with mortgage | 3.2 | 3.1 |  |
| Rent and other | 15.1 | 9.1 |  |
| **Number of ADL and IADL difficulties.** |  |  | 0.119 |
| **None** | 77.8 | 79.5 |  |
| **1** | 11.9 | 10.6 |  |
| **2+** | 10.3 | 9.8 |  |
| **Long term health condition** |  |  | 0.411 |
| No | 23.2 | 23.3 |  |
| An emotional, nervous or psychiatric problem | 1.3 | 1.0 |  |
| Other long-term health condition | 75.5 | 75.7 |  |
| **Living arrangement** |  |  | 0.034 |
| With an adult | 18.8 | 17.5 |  |
| Single person household | 17.2 | 15.6 |  |
| With partner aged 70+ only | 51.2 | 53.7 |  |
| With partner younger than 70+ only | 12.8 | 13.3 |  |
| **Receipt of practical help after the pandemic** |  |  | 0.728 |
| No | 35.2 | 34.8 |  |
| Yes | 64.8 | 65.2 |  |
| **Change in receiving practical help** |  |  | 0.020 |
| No change | 53.1 | 53.7 |  |
| Increase | 40.8 | 40.9 |  |
| Decrease | 3.7 | 2.5 |  |
| **Pre-pandemic emotional support** |  |  | 0.120 |
| A lot | 19.6 | 18.9 |  |
| Some | 32.7 | 31.3 |  |
| A little | 26.3 | 27.7 |  |
| None | 21.4 | 22.1 |  |
| **Change in emotional support** |  |  | 0.272 |
| About the same | 17.2 | 16.6 |  |
| More | 74.7 | 75.9 |  |
| Less | 8.1 | 7.5 |  |
| **Pre-pandemic contact score, mean (SD)** | 11.9 (3.5) | 11.9 (3.4) | 0.152 |
| **Contact score after the pandemic, mean (SD)** | 10.2 (3.6) | 10.2 (3.6) | 0.554 |
| **Financial transfer after the pandemic** |  |  | 0.639 |
| No | 85.5 | 85.2 |  |
| Yes | 14.5 | 14.8 |  |
| **Partner relationship change (closeness)** |  |  | 0.850 |
| No change or not in relationship | 91.6 | 91.5 |  |
| Better than before | 7.2 | 7.4 |  |
| Worse than before | 1.2 | 1.1 |  |

Source: authors’ analysis, Understanding Society: COVID-19 Study, 2020-2021.

* p-value for comparison between participants in the wave1 sample who did and did not respond to follow-up.

Note: All proportions are weighted using sample weights. Number of respondents are unweighted. Mean age difference test used ANOVA F test, others used Pearson Chi-Square test.

Supplementary Table 2. Cumulative hazard and 95% CI for loneliness over time.

| **Respondent Characteristics** | | **Cumulative hazard of loneliness % (95% CI)** | | | | | | |
| --- | --- | --- | --- | --- | --- | --- | --- | --- |
|  |  | **April 2020** | **May 2020** | **June 2020** | **July 2020** | **September 2020** | **November 2020** | **January 2021** |
| Total |  | 13 (12-13) | 19 (18-20) | 22 (21-23) | 25 (24-26) | 28 (26-29) | 33 (31-34) | 36 (34-37) |
| Gender | Women | 19 (18-21) | 28 (26-30) | 31 (30-33) | 37 (35-39) | 40 (38-42) | 45 (43-48) | 51 (48-53) |
|  | Men | 8 (7-8) | 12 (11-13) | 15 (14-16) | 17 (16-18) | 18 (17-20) | 23 (21-24) | 25 (23-26) |
| Living arrangements | Live alone | 28 (26-31) | 38 (35-41 | 42 (38-45) | 48 (45-52) | 52 (48-56) | 59 (55-64) | 67 (62-72) |
|  | Live with a partner only | 9 (8-9) | 15 (14-16) | 17 (16-18) | 20 (19-21) | 22 (21-23) | 26 (25-28) | 29 (27-30) |
|  | Live with someone | 15 (13-18) | 19 (16-22) | 22 (19-26) | 23 (20-27) | 31 (27-35) | 35 (31-40) | 38 (34-43) |
| Pre-pandemic emotional support | A lot | 25 (23-27) | 35 (32-38) | 39 (36-42) | 43 (40-47) | 47 (44-51) | 54 (50-58) | 59 (55-64) |
|  | Some | 11 (10-12) | 17 (16-19) | 21 (19-23) | 25 (23-27) | 28 (26-30) | 33 (31-35) | 36 (34-39) |
|  | A little | 11 (10-13) | 17 (16-19) | 20 (18-21) | 24 (22-26) | 25 (23-27) | 31 (29-34) | 33 (31-36) |
|  | None | 6 (5-8) | 11 (9-12) | 12 (11-14) | 14 (12-15) | 15 (13-16) | 17 (16-19) | 19 (18-22) |
| Post-pandemic emotional support change | More | 20 (18-23) | 28 (26-31) | 32 (29-35) | 37 (34-41) | 42 (38-45) | 48 (45-53) | 56 (52-61) |
|  | About the same | 10 (10-11) | 16 (15-17) | 18 (17-19) | 21 (19-22) | 23 (21-24) | 27 (26-28) | 29 (28-31) |
|  | Less | 21 (18-25) | 30 (26-35) | 41 (36-46) | 51 (45-57) | 52 (47-59) | 60 (53-67) | 62 (55-69) |
| Pre-pandemic contact score | Lower than the mean score | 9 (9-10) | 16 (14-17) | 18 (17-20) | 21 (20-22) | 22 (21-24) | 27 (25-29) | 30 (29-32) |
|  | Higher than the mean score | 16 (15-17) | 23 (21-24) | 26 (24-27) | 30 (28-31) | 33 (31-35) | 38 (36-40) | 41 (39-43) |
| Post-pandemic contact score change | More | 12 (10-13) | 17 (15-19) | 19 (18-22) | 23 (21-26) | 26 (21-26) | 30 (27-32) | 34 (31-37) |
|  | About the same | 11 (9-13) | 22 (19-24) | 25 (22-28) | 27 (24-30) | 30 (27-33) | 31 (28-35) | 34 (30-38) |
|  | Less | 13 (12-14) | 19 (18-21) | 22 (21-24) | 26 (24-27) | 28 (26-29) | 34 (32-36) | 37 (35-38) |
| Post-pandemic Practical help receipt change | Increase | 16 (14-17) | 22 (21-24) | 25 (24-27) | 29 (27-31) | 33 (31-35) | 39 (37-42) | 42 (40-45) |
|  | No change | 10 (9-11) | 16 (15-17) | 18 (17-20) | 21 (20-23) | 22 (21-24) | 27 (25-28) | 29 (28-31) |
|  | Decrease | 25 (19-32) | 29 (23-37) | - | 34 (26-42) | - | 38 (30-48) | 38 (30-48) |
| Post-pandemic financial transfer | No | 12 (11-13) | 18 (17-19) | 21 (20-22) | 24 (22-25) | 25 (24-27) | 30 (29-32) | 33 (32-34) |
|  | Yes | 16 (14-18) | 24 (22-27) | 28 (25-31) | 34 (31-37) | 39 (36-43) | 44 (41-48) | 50 (46-54) |
| Post-pandemic partner relationship change | Better than before | 10 (8-13) | 15 (12-18) | 20 (17-24) | 21 (18-25) | - | 29 (25-33) | 30 (26-35) |
|  | Not change or not in relationship | 13 (12-13) | 19 (18-20) | 22 (21-23) | 25 (24-26) | 27 (26-29) | 32 (31-34) | 35 (34-37) |
|  | Worse than before | 33 (24-46) | 73 (57-94) | - | 90 (70-100) | - | - | 90 (70-100) |

Source: authors’ analysis, Understanding Society: COVID-19 Study, 2020-2021.

N=8,645 observations

Supplementary Table 3. Adjusted odds ratios of new occurrence of loneliness during the pandemic among people aged 70+ (N=8, 645 observations)

| **Respondent Characteristics** | **Model 1**  **(Main effects)** | | **Model 2**  **(with interaction terms)** | |
| --- | --- | --- | --- | --- |
|  | **OR** | **95% CI** | **OR** | **95% CI** |
| Living arrangement (ref: With an adult) |  |  |  |  |
| Single person household | **1.81**ǂ | **0.99-3.32** | 1.93 | 0.77-4.85 |
| With partner aged 70+ only | **0.51*** | **0.30-0.87** | **0.33**** | **0.15-0.69** |
| With partner younger than 70+ only | 0.62 | 0.31-1.26 | **0.40*** | **0.16-0.99** |
| Receipt of practical help after the pandemic No (ref: No) |  |  |  |  |
| Yes | 1.30 | 0.94-1.81 | **1.32ǂ** | **0.95-1.84** |
| Change in receiving practical help (ref: No change) | | | | |
| Increase | 1.10 | 0.68-1.77 | 1.12 | 0.70-1.81 |
| Decrease | 1.11 | 0.31-4.00 | 1.13 | 0.32-4.04 |
| Pre-pandemic emotional support (ref: A lot) |  |  |  |  |
| Some | **0.34***** | **0.19-0.60** | **0.32***** | **0.18-0.57** |
| A little | **0.44**** | **0.24-0.80** | **0.43**** | **0.23-0.78** |
| None | **0.20***** | **0.10-0.42** | **0.19***** | **0.09-0.40** |
| Change in emotional support (ref: About the same) |  |  |  |  |
| More | **2.60**** | **1.50-4.50** | **2.62**** | **1.51-4.54** |
| Less | **6.57***** | **3.18-13.54** | **6.79***** | **3.28-14.04** |
| Pre-pandemic contact score | 1.04 | 0.97-1.12 | 1.04 | 0.96-1.11 |
| Contact score after the pandemic | 0.99 | 0.94-1.05 | **0.99** | 0.94-1.04 |
| Financial transfer after the pandemic (ref: No) |  |  |  |  |
| Yes | 1.17 | 0.85-1.61 | 1.15 | 0.84-1.59 |
| Partner relationship change (closeness) (ref: No change or not in relationship) |  |  |  |  |
| Better than before | **0.46**ǂ | **0.19-1.11** | **0.44**ǂ | **0.18-1.08** |
| Age group (ref: 70-74) |  |  |  |  |
| 75-79 | 1.03 | 0.64-1.65 | 1.03 | 0.64-1.66 |
| 80+ | 0.76 | 0.40-1.44 | 0.73 | 0.38-1.40 |
| Gender (ref: Men) |  |  |  |  |
| Women | **3.21***** | **2.04-5.05** | 1.74 | 0.64-4.74 |
| Housing tenure (ref: Own outright) |  |  |  |  |
| Owned with mortgage | 0.50 | 0.14-1.80 | 0.47 | 0.13-1.71 |
| Rent and other | 1.50 | 0.65-3.43 | 1.48 | 0.65-3.39 |
| Number of ADL and IADL difficulties (ref: None) | | | | |
| 1 | **1.80ǂ** | **0.94-3.43** | **1.78ǂ** | **0.93-3.40** |
| 2+ | **2.09ǂ** | **0.93-4.67** | **2.03ǂ** | **0.90-4.56** |
| Long term health condition (ref: No) |  |  |  |  |
| An emotional, nervous or psychiatric problem | 1.71 | 0.45-6.46 | 1.76 | 0.48-7.24 |
| Other long-term health condition | 1.43 | 0.91-2.24 | 1.42 | 0.94-2.29 |
| Month (ref: April) |  |  |  |  |
| May | 0.95 | 0.67-1.35 | 0.95 | 0.67-1.35 |
| June | 0.75 | 0.52-1.07 | 0.75 | 0.52-1.07 |
| July | 0.93 | 0.65-1.32 | 0.93 | 0.65-1.32 |
| September | 0.74 | 0.52-1.06 | 0.75 | 0.52-1.07 |
| November | **2.05***** | **1.42-2.95** | **2.07***** | **1.43-2.98** |
| January | **2.37***** | **1.65-3.41** | **2.40***** | **1.66-3.45** |
| Living arrangement # gender |  |  |  |  |
| Women# Single person household |  |  | 1.04 | 0.31-3.53 |
| Women # With partner aged 70+ only |  |  | 2.31 | **0.77-6.20** |
| Women # With partner younger than 70+ only |  |  | **3.55**ǂ | **0.81-16.42** |
| Variance level respondents | 10.38 | 8.31-12.97 | 10.75 | 8.60-13.44 |
| Model fit Log likelihood | -2204.46 |  | -2201.79 |  |
| LR test vs. logistic model: P value | **<0.001** |  | **<0.001** |  |

Source: authors’ analysis, Understanding Society: COVID-19 Study, 2020-2021.

***p<0.001;**p<0.01;*p<0.05; ǂp<0.1
